# Role of video-assisted thoracoscopic sympathectomy in the treatment of primary hyperhidrosis

**DOI:** 10.1590/S1516-31802003000500003

**Published:** 2003-09-01

**Authors:** Luiz Eduardo Villaça Leão, Renato de Oliveira, Renuzza Szulc, Jair de Jesus Mari, Pedro Luis Reis Crotti, José Julio Saraiva Gonçalves

**Keywords:** Hyperhidrosis, Thoracoscopy, Sympathectomy, Hiperidrose, Toracoscopia, Simpatectomia

## Abstract

**CONTEXT:**

Essential hyperhidrosis is a frequent disorder causing significant functional impairment. The advent and development of video-assisted thoracoscopic techniques now allows thoracic sympathectomy to be carried out precisely and safety with good results and minimal morbidity.

**OBJECTIVE:**

To assess the impact of video-assisted thoracic sympathectomy in patients diagnosed as presenting severe and disabling hyperhidrosis.

**TYPE OF STUDY:**

This was a longitudinal study of the clinical course of all hyperhidrosis cases selected for surgery between May 1999 and January 2003.

**SETTING:**

Division of Thoracic Surgery, Universidade Federal de São Paulo (UNIFESP).

**PARTICIPANTS:**

743 patients with surgery indicated due to palmar hyperhidrosis (49.8%), palmar-axillary hyperhidrosis (38.1%), craniofacial hyperhidrosis (8.9%) or isolated axillary hyperhidrosis (2.8%).

**PROCEDURES:**

Video-thoracoscopic sympathectomy was performed, isolating the second thoracic ganglion (T2) in all patients, with additional sympathectomy of T3 and T4 if necessary.

**MAIN MEASUREMENTS:**

The clinical course was followed up via questionnaires, phone calls, letters and statements. Simple questions were asked regarding the disappearance of symptoms and presence and intensity of compensatory sweating.

**RESULTS:**

The surgery was regarded as efficient in all cases of palmar hyperhidrosis. In the craniofacial hyperhidrosis cases, partial recurrence of the symptoms occurred in 2 cases (3.0%). Partial recurrence or persistence of symptoms occurred in 20% of the patients with predominantly axillary symptomatology. The compensatory sweating was considered disagreeable or uncomfortable by about 30% of the patients, but it only reached the level of regretting the operation for 3% of them. This occurred more frequently in patients with axillary hyperhidrosis. Ten cases of complications occurred.

**CONCLUSION:**

Thoracoscopic sympathectomy provides very good results in most patients, with a very low complication rate. However, the assessment of surgical results using conventional methods is imprecise and inaccurate. Different methodology, including quality of life assessment, must be used for comparing results and providing objective data on the results of this operation.

## RESUMO

**CONTEXTO:**

A hiperidrose essencial é afecção freqüente, que causa importante comprometimento funcional nos portadores. O advento e evolução das técnicas videotoracoscópicas permitiu que a simpatectomia torácica pudesse ser realizada de forma bastante precisa, segura, possibilitando bons resultados e mínima morbidade.

**OBJETIVO:**

Avaliar o impacto da simpatectomia torácica por vídeo-toracoscopia em pacientes portadores de hiperidrose severa e com importante comprometimento funcional.

**TIPO DE ESTUDO:**

Estudo de curso clínico longitudinal, com alocação de todos os casos de hiperidrose submetidos a tratamento cirúrgico entre maio de 1999 e janeiro de 2003.

**LOCAL:**

Disciplina de Cirurgia Torácica, Universidade Federal de São Paulo (Unifesp).

**PARTICIPANTES:**

Foram estudados 743 pacientes. A indicação cirúrgica foi principalmente hiperidrose palmar (49,8%); ou palmar-axilar (33,1%). A hiperidrose crânio-facial foi critério de indicação em 8,9% e a hiperidrose axilar isolada, em 2,8%.

**PROCEDIMENTOS:**

A operação foi realizada através de simpaticotomias tronculares tranversas, isolando-se o segundo gânglio torácico (T2) em todos os pacientes, sendo realizadas simpaticotomias adicionais sobre T3 ou T4 em função dos sintomas.

**VARIÁVEIS ESTUDADAS:**

A evolução clínica foi obtida através de questionários, telefonemas, cartas e depoimentos. Foram formula-das questões simples, relacionadas ao desaparecimento dos sintomas e à presença e intensidade do suor compensatório.

**RESULTADOS:**

A operação foi eficaz para a resolução da hiperidrose palmar na totalidade dos casos. Nos casos de hiperidrose crânio-facial, ocorreu recidiva parcial dos sintomas em dois casos (3,0%). Nos casos de hiperidrose axilar predominante, ocorreu recidiva parcial ou persistência de sintomas em 20% dos pacientes. O suor compensatório era considerado desagradável para cerca de 30% dos pacientes, porém em apenas 3% deles chegou ao nível de arrependerem-se da operação. Esse fato ocorreu com maior freqüência em portadores de hiperidrose axilar. Dez casos de complicações ocorreram.

**CONCLUSÕES:**

A simpatectomia torácica por vídeo-toracoscopia propicia resultados muito bons na maioria dos pacientes, com um baixo índice de complicações. Contudo, a avaliação dos resultados cirúrgicos pelos critérios convencionais é inadequada, subjetiva e imprecisa. Há necessidade de metodologia diversa, incluindo avaliação de qualidade de vida, para que se possam comparar resultados cirúrgicos.

## INTRODUCTION

Primary or essential hyperhidrosis is a disorder characterized by excessive sweating in disproportion to that required for thermoregulation and dissipation of body heat. In most cases this excessive sweating is aggravated by emotional factors and also by heat. Hyperhidrosis presents preferential sites such as head and face, palms, soles and axillae, in addition to their various associations. However, the severe clinical characteristic of hyperhidrosis is the intense discomfort it causes to the patient. This discomfort can be seen in a great number of routine activities, leading to significant unease, embarrassment and shame, and severely compromising the affective, professional and social life of those affected. The etiology of this dysfunction is not completely known yet, but it is certain that there is excessive sympathetic stimulation by the sudomotor center.

Copious palmar sweating causes difficulty in social contact, writing, manual activities, car driving and handling objects, among others. Sweaty feet, besides the discomfort they produce, render the use of sandals or even walking barefoot difficult. Axillary hyperhidrosis dampens and stains clothes in addition to embarrassing their wearers, who usually then use only black or white clothes. Cranio-facial hyperhidrosis intensely embarrasses those who present it, by drawing attention to them and at the same time making them feel insecure, afraid and lacking in confidence. There is no doubt that there is clinical predominance of varying intensities of embarrassment, isolation, insecurity and difficulties in the sufferers’ social, professional and affective lives. In a significant number of cases this insecurity is aggravated by the little importance given to the patient's complaint, both by relatives and even by the attending physicians, failure in the diagnosis and often a succession of previously proposed ineffective measures.

For many years it has been known that cervicothoracic sympathectomy can eliminate palmar hyperhidrosis symptoms. The presence of serious complications after conventional surgery, especially Horner's syndrome caused by stellate ganglion injury, has led this procedure to be little used in the treatment of hyperhidrosis.^1^ In the 1950s, Edhard Kux^2^ used direct thoracoscopy to successfully perform thoracoscopic sympathectomy, with the potential ability to avoid such complications. However, this important advance did not have the expected impact probably because minimally invasive surgeries at that time were not popular, nor did they receive attention from the medical community. In the 1990s, the systematization of video-assisted thoracoscopy allowed thoracic sympathectomy to be indicated and used with significant benefit to patients. Thoracoscopic sympathectomy is indicated in different situations, notably hyperhidrosis, reflex sympathetic dystrophy, ischemic upper limb syndromes and long QT syndrome.

Good results using thoracoscopic sympathectomy have been presented by our group,^3^ as well as in other Brazilian reports,^4^ although hyperhidrosis represented a small proportion of the cases. Upon reviewing the literature, the extremely small number of hyperhidrosis cases undergoing operation in Brazil becomes evident, in comparison with other countries. We believe that this is probably due to insufficient knowledge of the disease among our colleagues (maybe due to the huge clinical interface involved) as well as among patients themselves.

The initial experience with such cases was published in 1999 and 2000.^5-7^ From the outset, the enormous satisfaction of hyperhidrosis patients undergoing operation has been noteworthy. There has been a great flow of patients, many of whom without previous diagnosis or attributing their condition to “anxiety”, “this is my nature”, “weakness” or even “dirtiness” (usually a depreciative situation), as well as the characteristic nuisance in everyday life. Such unexpected incidence seems to show that this disorder, which is so frequently without diagnosis, may present higher prevalence than we had supposed. At the same time, this increase in experience has fostered several modifications and simplified the surgical technique, which has become quicker, safer and cheaper.^8^ The aim of the present study was to assess the outcomes among patients presenting severe and disabling hyperhidrosis who were submitted to video-assisted thoracic sympathectomy.

## METHODS

During the period between April 1999 and January 2003, 743 patients with primary hyperhidrosis were operated on. Their ages ranged from 9 to 59 years, with a mean of 25.4 years. With regard to distribution according to sex, female cases predominated, with 453 female patients (61.0%) and 290 male patients (39.0%).

The criteria for surgical indication regarding the subtypes of hyperhidrosis are shown in Table 1. It should be mentioned that the above numbers do not correspond to the real incidence of patients who were seeking a surgical solution. In our experience, cases of isolated or very predominant axillary hyperhidrosis have not been indicated for surgical treatment. On the other hand, more patients with craniofacial hyperhidrosis have been accepted for surgical treatment.

**Table 1:** Indication for surgery in relation to the predominant hyperhidrosis sites (regarded as those that were the most annoying and uncomfortable for the patient)

Hyperhydrosis site	No. of patients	Percentage
Facial blushing	3	0.4%
Craniofacial hyperhidrosis with or without facial blushing and associations	66	8.9%
“Pure” axillary hyperhidrosis	21	2.8%
Palmar-axillary-plantar hyperhidrosis	283	38.1%
Palmar-plantar hyperhidrosis	370	49.8%
**Total**	**743**	**100%**

## SURGICAL TECHNIQUE

After induction of anesthesia the patients were intubated using simple tracheal catheters or double-lumen Robertshaw (Bronchocath) catheters. When the Robertshaw catheter was utilized, bronchoscopic control was preferentially used in the positioning of the catheter. During the study of these cases, the use of double-lumen catheters was slowly discontinued in favor of the use of simple orotracheal catheters. During the intrathoracic time, ventilation was interrupted in that lung by apneic maneuvers and hypoventilation (double-lumen catheter) or by apneic oxygenation (simple orotracheal catheter).

Positioning of the patient was the critical point in improving the technique. Lateral decubitus was substituted for dorsal decubitus with elevation of the trunk and the upper limbs in an abducted position, taking extreme care regarding protection of the vascular structures and the brachial plexus. Elastic stockings were frequently used to minimize the decrease of venous return in the half-sitting position.

Bronchoscopes of 4 mm to 5 mm were used in the axillary region, with zero degrees and two orifices. In our systematization, the right side was always the first to be approached. We used between 4 mm (permanent) and 5.5 mm (disposable) portal diameters in the axillary region. The two orifices could be placed in the hairy region of the axillae. The first portal was usually plastic and allowed the entry of air and pulmonary collapse. The second portal, usually metallic, was then positioned under direct viewing.

Upon pulmonary collapse, the visualization of the pleural cavity apex was quite adequate. The ribs were carefully identified, making sure that the first rib was viewed in a completely different plane and direction from the others. Thus, identification of the second rib was critical and constituted the most important repair point. The second thoracic ganglion (T2) was usually located in the space between the second and third ribs, sometimes over the rib head and sometimes over the costovertebral articulation or even over the trans-verse process of the vertebra.

The sympathetic trunk was often identified visually and, if not, by palpation or dissection. A multiplicity of anatomical variations and also those depending on age, amount of subpleural fat, sex and constitution, etc, were always considered. In most cases, sectioning of the sympathetic trunk over the second and third rib was used, thereby isolating the T2 and T3 fibers. In several cases, an incision over the fourth rib was also made in order to isolate T4 (particularly in cases of axillary hyperhidrosis). The incision, made using a 4.5-mm hook connected to a ValleyLab electrocauterizer, was usually extended laterally so that Kuntz fibers could also be sectioned.

After completing the isolation of sympathetic ganglia and confirming perfect hemostasis, we used a 14 or 16-F catheter for air removal, using a water seal system during pulmonary re-expansion and the Valsalva maneuver. After this procedure, an absorbable stitch (Monocryl) or acrylic glue (Derma-bond) was used.

The procedure was repeated on the left side, with special attention to the protection of important exposed vascular structures (descending aorta and subclavian artery). In cases where significant adherences occurred and a bloody pleural area or even a small alveolar fistula remained, we chose to keep the same plastic catheter and connect it to a conventional thoracic drainage system. We observed a much higher incidence and intensity of pleural adherences in the right hemithorax. Sometimes, when complete obliteration of the pleural space occurred due to adherences, we opted for the use of a single 10-mm portal and 10 x 5 scope with a working channel. A blunt or cutting dissector (EndoPeanut) or even an electric scalpel could be used through the working channel.

Coagulation using a monopolar electronic scalpel was used sparingly in view of the proximity of the spinal medulla. Special forceps adapted to the bipolar scalpel were used in some cases. A harmonic scalpel with a 5-mm endoscopic hook-shaped tip was used to perform sympathectomy in the last 88 consecutive patients (11.8%) of the series. After concluding the surgery, chest x-ray was performed while awaiting the end of anesthetic drug action. Once the surgery was finished, the patients were directed to the post-anesthesia recovery room.

The chest tube was left in place only when there was air leakage, which occurred usually when dense adhesions were present. Patients were operated in the mornings and we elected to discharge uneventful patients the following morning (even though most of them could have been discharged earlier). When a chest tube was needed, the patient was kept in the hospital for a longer period (one to three days). Patients were carefully evaluated by the surgical team, and most postoperative complications, when present, were diagnosed in the recovery room (even brachial plexopathy and Horner syndrome). Compensatory sweating is a crucial symptom to be evaluated during medium to long-term follow-up. However, the difficulty in interpreting the intensity of sweating, both by the patient and the examiner, is noteworthy.

Although the follow-up was not standardized, all patients were asked about any continuing impact of previous symptomatology, and any compensatory sweating. Most patients did not come into the clinic regularly for attendance. Contact with patients was carried out by means of phone calls, fax, internet, and letters. All subjects received the following core questions: How is your hand? Are you still sweating anywhere? Is there any compensation going on? How does it disturb you? Is the compensatory sweating bothering you? How do you feel about the surgery? Do you regret the surgery? All subjects were followed up for at least three months.

## RESULTS

Upon recovering consciousness in the surgical or recovery room, the patients observed that their hands were warm and dry. Thus, patients with palmar and craniofacial hyper-hidrosis were already presenting good results in the immediate postoperative period. Table 2 summarizes the relevant complications observed in these patients.

**Table 2:** Severe postoperative complications after bilateral thoracic sympathectomy in relation to hyperhidrosis subtype

Hyperhidrosis subtype	No. of patients	Complications No. of patients
**Facial blushing**	3	none
**Craniofacial with or without associations**	66	Partial recurrence – 2* Horner syndrome – 1
**“Pure” axillary**	21	Recurrent symptoms – 4 DCS – 3
**Palmar-axillary**	283	DCS – 1 Horner syndrome – 1 Late hemothorax – 1 Late chylothorax – 1
**Palmar**	370	DCS – 1 Horner syndrome – 2 Late hemothorax – 2
**Total**	**743**	

*DCS – disabling compensatory sweating leading to regret for surgery.*

* *patients now without symptoms and under treatment with oxybutynin.*

It was possible to perform bilateral sympathectomy in all cases in which an operation was proposed. In no instance was the operation not accomplished due to adhesions, bleeding, etc. In 18 patients (2.4%), the chest tube was left in place for one to three days.

In all patients with palmar symptoms, therefore, the result was clinically significant. No relapses of palmar hyperhidrosis occurred during the assessed period (a follow-up period of between three months and three years).

In two patients (2/60; 3.3%) with cranio-facial hyperhidrosis who were operated on at the beginning of the series, partial recurrence of symptoms occurred. In both patients the symptoms were controlled using anticholinergic medication and there was no need for reoperation. In the other patients with cranio-facial hyperhidrosis or facial blushing, no relapses occurred.

The immediate result in the axillary region was fairly good, but it seemed to worsen with time. Regarding the axillae, relapses or persistence of less intense symptoms occurred, on at least one side, in approximately 15 to 20% of the axillary hyperhidrosis cases.

Approximately 70% of the patients reported a significant decrease in excessive plantar sweating (sweating only under intense heat). The remaining 30% did not present a significant decrease in the plantar symptom.

Horner syndrome was observed in four cases (0.54%). However, in three of these patients the syndrome reverted within a period of eight months after the surgery (the remaining case was only operated two months before the writting of this report). The chest apex was obliterated by adhesions for all these patients, and in one case there was also the presence of a cervical rib.

Brachial plexopathy occurred in two patients (0.27%), and was bilateral in one and unilateral in the other. Recovery was complete in both cases, occurring after approximately two weeks (unilateral case) and three months (bilateral case). In this last case, with bilateral and asymmetric impairment, the patient probably had a predisposing clinical condition (pressure sensitive neuropathy).

In three cases (0.40%), late pleural effusion (hemothorax) occurred when patients had already resumed their usual activities (between one and two weeks after the surgery). Thoracocentesis was sufficient for resolution in these three cases. Chylothorax occurred in one case (0.13%) approximately two weeks after surgery. This case was treated with thoracocentesis and dietary measures were recommended.

Neuralgic pain and/or paresthetic sensations at the thoracic wall occurred rather frequently, particularly in women over 35 years old. To measure pain was not an objective of this research. However, we noted that these symptoms were well tolerated by an absolute majority of the cases, and required no treatment. This pain was only an important symptom for six patients (0.8%), and it was treated by pain specialists using tricyclic antidepressants and other anti-epileptic drugs. The symptomatology subsided completely within a maximum of three months. The study and prevention sequence for these painful symptoms after surgery are the subject of research to be published soon.

When “postoperative complications” are mentioned in sympathectomy, we believe that compensatory sweating is the most relevant and important postoperative symptom. This phenomenon is also called reflex hyperhidrosis, and patients were always given information about it before surgery. Its intensity is not always totally predictable, but the phenomenon was reported by approximately 70% of the patients. There is a tendency for this symptom to diminish over a period of one year or a little more after surgery, thereafter usually persisting at a much lower level. We observed overall that although constituting a disturbance or “discomfort” in 25 to 30% of the patients, only 3%, or 22 patients, considered it “unbearable”. A smaller number among these 22, five patients, expressed the opinion that they considered this phenomenon to be sufficiently severe to make them regret having undergone the operation. It should be noted that, in patients with axillary hyperhidrosis, complaints regarding the severity of compensatory sweating were more frequent. However, the mention about this discomfort and its characterization was totally spontaneous; therefore it can be considered a subjective notion of a side effect of the surgery, rather than a measure of a complication.

## DISCUSSION

Sweating is required for the control of body temperature, especially during exercise or at higher ambient temperatures. Excessive sweating is regulated by the sympathetic autonomous nervous system. Hyperactivity of the sweat glands leads to excessive sweating. This condition is known as hyperhidrosis.

Hyperhidrosis is a relatively frequent phenomenon, with a reported incidence of approximately 1% of the population.^9^ It is not a severe, life-threatening disease, but represents an extremely uncomfortable situation causing deep social embarrassment and relationship and psychological disorders in such patients, who frequently becomes socially isolated and acquire habits that attempt to hide the problem. Curiously, and for a variety of reasons, the proportion of such patients that have their problem solved and efficiently and lastingly treated is insignificant.

With the advent of video-assisted thoracoscopy, the spread of this method and its safety, thoracoscopic sympathectomy has acquired an important position in the treatment of this disease, especially in Asia and Europe.^10-22^ This access route, after the surgeon has acquired adequate experience through training in video-assisted thoracoscopy, allows well-directed surgery to be performed, with minimal risk of the disagreeable complications that occurred in the past when the transcervical route was used, as well as the sequelae and scars of classical thoracotomy.^23-26^

In using the transcervical route, injury to the stellate ganglion is in fact very frequent and expected, producing significant sequelae: the so-called Claude-Bernard-Horner syndrome (palpebral ptosis, miosis and enophthalmos). In addition, injuries to the phrenic nerve and direct injury to the brachial plexus, among others, may occur. Such an incidence of complications led this procedure to be very little used in hyperhidrosis, and was restricted to those patients with severe ischemia.^27^

The simplicity of video-assisted thoracos-copy, allowing precision and reliability of access to the sympathetic trunk, has turned this procedure into a very good alternative for thoracic sympathectomy in relation to the whole spectrum of indications. Among the classic indications, such as ischemia, Raynaud's phenomenon, causalgia (reflex sympathetic dystrophy) and hyperhidrosis itself, others can be cited, which in the same way as hyperhidrosis, cause embarrassment and discomfort to patients, such as excessive facial blushing, which may or may not be associated with craniofacial hyperhidrosis.^28^

**Figure 1:**
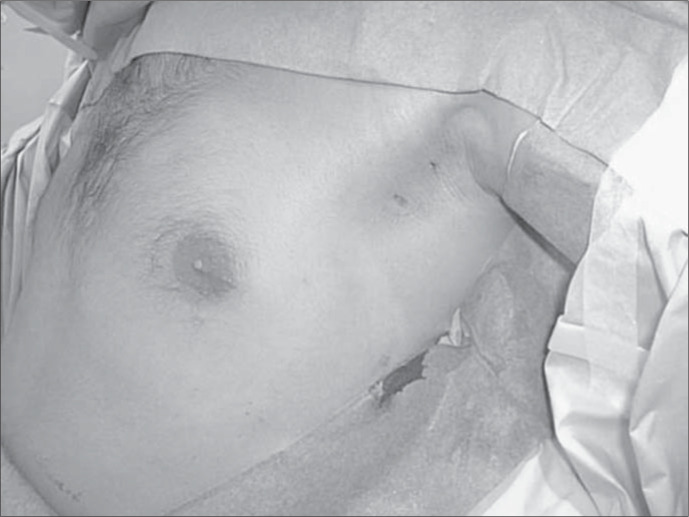
Male patient at the end of the procedure. Final appearance: note positions of the incisions in the hairy area of the axilla. The incisions were closed using surgical glue (Dermabond).

**Figure 2:**
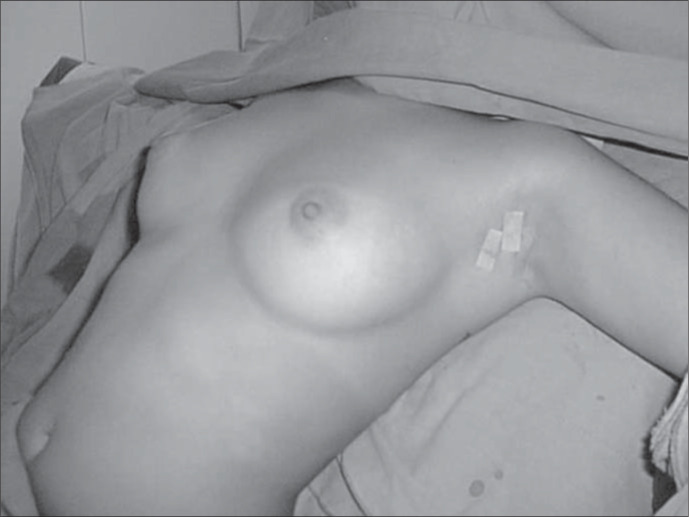
Female patient. Final appearance: note positions of the two small incisions, here closed using adhesive skin closure tape.

The video-assisted thoracoscopic procedure standardized initially by most surgeons was resection of the sympathetic trunk, from the lower part of the stellate ganglion (T_1_) to T_4_.^9,13^ In the beginning, 10-mm scopes and several instruments were used. Thoracic drainage was mostly used and many surgeons performed the bilateral operation in two different surgical sessions. With advances in the method and greater skills developed among surgeons, the operation came to be performed in an even more simplified manner. In some centers, the surgery is performed using two punctures through which a 2-mm scope and the tip of a cauterizer are introduced with the objective of selectively destroying the second thoracic ganglion (T_2_).^10,12,14-16,18-20^ For this strategy, CO_2_ insufflation is required in order to deviate the lung. The scar so produced is minimal and the procedure is esthetically excellent, with a scar the size of a percutaneous puncture (needlescopic sympathectomy). In our group, the technique has also undergone modifications: we have been using only two 3 to 6-mm punctures and using 4 to 5-mm scopes. We consider that the esthetic results from our method have been quite satisfactory (Figures 1 and 2) and we have chosen not to follow the route of using 2-mm scopes because of the risk of endangering the results through the worse viewing conditions (finer scopes) and reduced options for handling and ablation of the sympathetic system (sectioning or cauterization). At the moment, we believe that the possible decrease (minimization) in scar size does not justify the risk that the postoperative results may be impaired.

The presence of postoperative complications in the present series remained at quite an acceptable level, which was comparable to a great number of cases published in detail.^2,29^ It should be noted, however, that different authors have report the incidence of complications in different ways, some referring to sympathectomy procedures performed and others to patients operated on. In the present series, we have focused on 743 patients who underwent 1,486 sympathectomy procedures.

Horner's syndrome was observed in four patients (0.54%), of whom three have already shown complete regression of the symptoms. Pleural adherences were present in all of these cases and in one of them there was also the presence of a cervical rib. We think that probably thermal or electric injury occurred next to the stellate ganglion.

Four patients showed pleural accumulations after surgery. In three patients (0.40%), hemothorax was found between one and two weeks after the operation (these three patients were treated using thoracocentesis alone). One patient presented severe thoracic pain upon effort after the tenth postoperative day, and chylothorax was observed (0.13%). He was submitted to thoracocentesis and simple dietary measures, which showed a good outcome.

It is important to emphasize that postoperative complications related to the positioning of the patient on the operating table should be viewed with extreme caution. The brachial plexus is very vulnerable to injuries, in any of the positions adopted by different surgeons: lateral decubitus, ventral decubitus or, as adopted in our series, dorsal decubitus (half-sitting position with upper limb abduction). Thus, attention should be paid to prophylaxis of the injuries by compression and distension of the brachial plexus, by the whole surgical and nursing team. In the two patients in whom such complications occurred (0.27%), recovery was complete within a period of 2 weeks in one patient (unilateral) and approximately 3 months in the other (bilateral and asymmetrical impairment, probably predis-posing to disease).

In the recent literature there is some controversy between the need for resection of the sympathetic trunk versus simpler and quicker procedures such as electrocoagulation, cryocoagulation, harmonic scalpel, destruction by radiofrequency, destruction by laser or interruption of the trunk by titanium clips. There is no evidence for superiority of one technique over another, and it can be said that all methods may be efficient for the destruction of the sympathetic trunk. In our group, for most cases we perform sectioning using monopolar electrocautery, so as to produce the least passage of electric and thermal energy over the sympathetic trunk, particularly for the section over the second rib.

Another option for performing sympathectomy is the harmonic scalpel, in which ultrasound vibration allows sectioning of the sympathetic trunk by vaporization, without significant increase in temperature and passage of electric current. The harmonic scalpel was used in 88 patients in this series (11.8%). The value of this resource, as well as cost/benefit assessments, will be a subject for study by our group.

In most cases, the innervation of upper limbs and axillary regions depends on the T2 to T5 ganglia. Thus, the surgery is usually conducted by targeting the T2 and T3 ganglia in the cases of palmar and palmar-axillary hyper-hidrosis (T4 is possibly also targeted when axillary symptoms are regarded as severe). It should be noted that the stellate ganglion (C8-T1) participates in the innervation of the upper limbs in approximately 10% of cases.

We emphasize also that most postganglionic sympathetic fibers responsible for face innervation originate from T2, which therefore justifies the ablation of this ganglion in the treatment of craniofacial hyperhidrosis.

Experience accumulated from this series of operated patients is being analyzed by our group and will be the subject of future publications with regard to technical modifications, quality of life, psychological aspects and also the surgical results in relation to the different sites. However, we deem it worthwhile to comment on some of our impressions, without prejudging a definitive assessment:

1. The results are very rewarding in relation to palmar hyperhidrosis, with the disappearance of the symptoms in all cases. Compensatory sweating is usually well-tolerated and the satisfaction index is quite high. A small number of patients may report slight dampening of the hands, to a much lower extent than before surgery. We believe that this fact is due to the small percentage of cases in which there is contribution of the T1 ganglion to sympathetic palmar innervation;
2. Craniofacial hyperhidrosis is usually a less frequent condition that appears in young adults but seems to produce more severe symptoms from a psychological point of view. The results from surgery are fairly good (success in over 95% of cases). However, patients should be advised that after surgery, in addition to compensatory sweating, they will have dry hands and should use humectant cream. Moreover, experience and good clinical criteria in the diagnosis and indication of craniofacial surgery are very important. Contrary to palmar hyperhidrosis, for which the diagnosis is readily made, in craniofacial hyperhidrosis differential diagnoses have to be considered (significant sweating without presenting the disease);
3. “Pure” axillary hyperhidrosis is the condition for which the surgical results we observed were poor. There was a higher index of relapse and symptom persistence, due to greater anatomical variation. In addition, compensatory sweating may be more severe, probably related to the larger denervated area. Thus, in such cases, other therapeutic options available should be considered, such as anticholinergics, botulinus toxin application and the different surgical methods for sweat gland resection in the axillary region.

Immediate results, i.e. the complete disappearance of hyperhidrosis, are the rule. In the few reported cases of symptom persistence, there was erroneous identification of T_2_^4^ or the presence of accessory Kuntz fibers. Another very frequently observed phenomenon is the substantial improvement or disappearance of plantar hyperhidrosis symptoms.^21,30^ The physiological mechanism for this improvement has not yet been explained clearly, but all authors, including us, have observed that such improvement may be seen in 70-80% of cases.

In discussing the thoracic sympathectomy results, the crucial point is the so-called “compensatory sweating”, in which the patient reports an increase in excessive sweating in other body regions, especially the back and abdomen. Attempts to eliminate compensatory sweating have been the subject of several studies by some authors, who have tried to avoid this side effect by modifying the procedure, without sectioning the sympathetic trunk and only sectioning the communicating branches and postganglionic fibers – the so-called Wittmoser procedure.^12^ These authors, however, observed that the decrease in compensatory sweating was not significant. Moreover, there was an absence of improvement in plantar symptomatology and higher incidence of palmar hyperhidrosis recurrence.

In assessing the results from thoracic sympathectomy, it is very easy to compare the incidence of postoperative complications between the different authors. Conversely, the most difficult and controversial point is how to assess compensatory sweating between different patients (which makes it difficult to compare different studies). In reviewing the literature, incidences ranging from 5% to 85% can be found. Considering “compensatory sweating” only as the increase in sweating in the trunk, this phenomenon would be expected to occur in almost all cases. At the other extreme, there are those patients who present this condition such that it not only causes discomfort, but also a situation of such unbearable intensity as to make the patient regret the surgery. Thus, in addition to compensatory sweating being an extremely subjective symptom, it also depends on the patient's preparation and information before the surgery, his/her psychological makeup and expectations, and even the temperature and humidity conditions in which he/she lives and works. It is reasonable to conclude that the most consistent assessment of the surgical result in hyperhidrosis – the postoperative satisfaction – depends on the patient him/herself and may only be objectively measured by assessment of the quality of life, through applying appropriate methodology.

Thus, considering the results obtained using video-assisted thoracoscopic sympathectomy, we may state that this is a minimally invasive and extremely efficient method for the treatment of hyperhidrosis. We believe that it can be considered as the only efficient method for curing moderate and severe hyperhidrosis of the hands and face. It is the method of choice, especially if other options have already been tested without satisfactory result. It also constitutes an efficient method for excessive facial blushing.^22^ It may additionally be used for axillary hyperhidrosis, albeit with long-term assessment and after comparison with the different “local” surgery techniques for sweat glands in the axillary region.^1,29,31,32^

## CONCLUSION

Our experience suggests that video-assisted thoracic bilateral thoracoscopic sympathectomy constitutes a valid and feasible intervention for the definitive treatment of palmar and craniofacial hyperhidrosis. However, we consider that we do not yet have adequate means for objectively assessing the most important results from this operation: improvement in the quality of life. Thus, we think that until such methods are developed, validated and applied, the physician's experience, common sense and sensitivity will still prevail. And furthermore, in complex cases, the real value of the surgical treatment will often depend on the surgeon's judgement on a case-by-case basis.
